# Childhood obesity and adolescent follow-up depressive symptoms: exploring a moderated mediation model of body esteem and gender

**DOI:** 10.1007/s00787-023-02348-9

**Published:** 2024-02-07

**Authors:** Lucia Beltrán-Garrayo, Junilla K. Larsen, Rob Eisinga, Jacqueline M. Vink, Miriam Blanco, Montserrat Graell, Ana Rosa Sepúlveda

**Affiliations:** 1 Department of Biological and Health Psychology, Faculty of Psychology, Autonomous University of Madrid, Madrid, Spain; 2 Behavioural Science Institute, Radboud University Nijmegen, Nijmegen, The Netherlands; 3 Department of Child and Adolescent Psychiatry and Psychology, University Hospital Niño Jesús, Madrid, Spain

**Keywords:** Childhood obesity, Adolescent depression, Body esteem, Mediation, Moderated mediation

## Abstract

Obesity is a well-recognized risk factor for adolescent depressive symptoms, but mediating mechanisms of this association have scarcely been studied. This study is unique in examining an indirect pathway of this link via body esteem (BE) prospectively from childhood (8–12 years) to adolescence (13–18 years). In addition, potential gender moderation was examined. This study utilized data from a case–control study comparing 100 children with and without obesity matched on important confounders (age, gender, and socioeconomic status). Our findings provide support for the mediating role of BE in the link between childhood weight status and adolescent depressive symptoms at a 5-year follow-up. This mediation effect did not differ between boys and girls. The findings suggest the relevance of specifically targeting children’s BE in preventive intervention programs among children with obesity to prevent future mental health problems.

**Supplementary Information:**

The online version contains supplementary material available at 10.1007/s00787-023-02348-9.

## Introduction

Depressive disorders are highly prevalent mental problems that rise sharply during puberty, especially for girls, and can seriously impact physical and psychological health [1, 2]. As depressive symptoms importantly underlie depressive disorders, there is a pressing need to identify and understand factors that contribute to the enhanced risk for adolescents’ depressive symptoms [3].

Recently, accumulating evidences have suggested a bi-directional association between depression and obesity [4, 5]. As such, obesity in childhood might be an eminent risk factor for the development of adolescent depressive symptoms [6–8]. This link is supported in adult populations by Mendelian randomization (MR) studies [9–11]. While results appear to be more inconsistent for children [7, 12], a recent MR study also suggests that childhood obesity is linked to the development of major depressive disorder during adulthood [13]. As such, identifying risk factors that mediate between obesity and depressive symptoms may provide a roadmap to prevent the escalating mental health consequences of childhood obesity.

### Body esteem-mediating effects

Body esteem (BE), defined as self-evaluation of one’s body or physical appearance [14], could be a prominent underlying mechanism explaining the development of obesity-linked depressive symptoms during adolescence [15, 16]. The subjective perception of the own body depends on the interplay of the person with his or her social context. Specifically, children who deviate from the thin ideal (e.g., children with obesity) are more likely to experience weight stigma, primarily through weight-related teasing [17]. It is thus not surprising that the literature has consistently found that children and adolescents with obesity tend to have lower BE than their normal-weight peers [18–20]. Moreover, body dissatisfaction is one of the most consistent predictors of depressive symptoms during adolescence [21–25]. Accordingly, some previous studies among both adolescent and adult populations have provided evidence for the conjecture that BE may mediate the obesity–depression link [26–32]. However, these studies are limited by their cross-sectional design. That is, they give no insight into the temporal order and developmental pathways of the findings. Our prospective study fills this prominent gap.

### Moderating effects of gender

There is evidence of a stronger link between obesity and depressive symptoms among females than males [6, 7, 32]. However, whether this might reflect a gender-specific mediation role of BE has been poorly studied. The study by Xie and colleagues [33] is, to our knowledge, the only one that has examined this potential gender-moderated mediation, and they did it among Asian and Hispanic adolescents. They found a significant mediation effect of BE on the association between being overweight and depressive symptoms among adolescent Asian girls. Regarding specific links, Shriver et al. [34] found that overweight was prospectively associated with BE only among girls, but when weight status reached obesity, the association with BE was significant for boys and girls. In addition, Ricciardelli et al. [18] reported no significant differences in the impact of weight status on BE between girls and boys. Concerning the BE to depressive symptoms pathway, some studies suggest that BE is a stronger predictor of depression in girls than in boys [24, 35, 36], while other studies suggest equivalent effects of BE on depressive symptoms in girls and boys [22, 37, 38]. Possible gender differences can be explained by the fact that boys generally place more importance on other domains than appearance, while appearance plays a prominent role in girls’ self-esteem and mental health [38]. However, since previous studies are scarce and mixed, further research is required to understand potential gender differences, particularly regarding BE prospective mediating effects.

## Current study

**Fig. 1:**
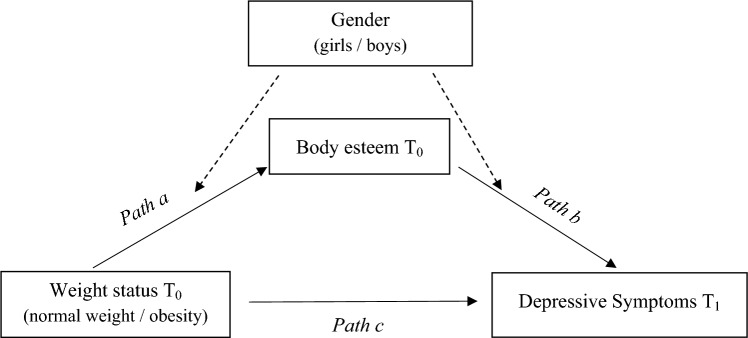
Diagram of moderated mediation model. Note that solid lines represent the direct and indirect (mediation) pathways; dotted lines represent the moderation effects in the indirect pathway (moderate mediation). Covariates (depressive symptoms *T*_0_, Age, and SES) are included in the analyses but are not displayed in the figure

The current study aims to cover the mentioned gaps by investigating whether BE is a mediator of the prospective childhood obesity–adolescent depressive symptoms pathway and whether mediation is different for boys versus girls (see Fig. 1). Based on prior considerations, our *first hypothesis* was that children with obesity would be concurrently and prospectively at higher risk for depressive symptoms, compared to children with normal weight. Our *second hypothesis* stated that, longitudinally, the childhood obesity group would develop more depressive symptoms after 5 years, compared to the normal-weight group, controlled for baseline depressive symptoms. The *third hypothesis* argued that BE would mediate the prospective association between childhood obesity and adolescent depressive symptoms. And in the *fourth hypothesis*, we hypothesized that the mediating effect of BE would be conditional on gender (i.e., moderated mediation), in the sense that the mediation effect will be stronger for girls than for boys. We made no further a priori hypothesis about the specific moderating role of gender in the obesity-to-body-esteem pathway. However, despite the mixed findings in the literature, we theoretically expected a stronger association for girls for the body esteem-to-depressive symptoms pathway.

## Method

The procedures and data analyses for this paper were pre-registered on the Open Science Framework (https://osf.io/dc67e). A minor deviation from the preregistration is discussed in “Strategy of analysis”.

### Participants and procedure

Participants were part of the “ANOBAS project” (PSI2011-23127), a case–control study to assess early risk factors for childhood obesity. The initial sample consisted of 100 children aged 8–12 years: 50 children with obesity (*Z*-BMI score ≥ 2) matched with 50 children with normal-weight (*Z*-BMI score ≥ −2 to < 1), according to age, gender and socioeconomic status [38]. The current study has a prospective two-wave design and compares these two groups on the development of depressive symptoms at the 5-year follow-up, when participants were 13–18 years old.

Data at baseline (*T*_0_) were collected between 2013 and 2016, while data at follow-up (*T*_1_) were collected between 2018 and 2021, in the region of Madrid, Spain. For *T*_0_ data collection, the obesity group was recruited from Primary Health Centers and the normal-weight group from 13 primary schools (matched 1:1). Details of the recruitment and sample selection procedure at *T*_0_ have been published elsewhere [39, 40]. At *T*_1_, participants were contacted by telephone or email to decide whether they wanted to participate in the follow-up, which was completely voluntary. The participation at each time point involved a semi-structured clinical interview with the child and at least one family member, a battery of self-reported questionnaires, and a measurement of weight and height. The assessments were carried out by the trained interviewers at the Niño Jesús Children’s Hospital, and the questionnaires were completed on paper. The study received ethical approval from the Niño Jesus Children Hospital and the Autonomous University of Madrid. Parents’ written consent and adolescents’ assent was obtained.

Out of 100 participants (see Supplementary Table S1), 70 participated in the follow-up (obesity group *n* = 34, normal-weight group *n* = 36). When comparing the cohort of children with obesity with the cohort of children with normal-weight that completed the follow-up, no significant differences were found in terms of age (*t* = 0.24, *p* > 0.05), gender (*χ*^2^ = 0.49, *p* > 0.05), and socioeconomic status (*χ*^2^ = 5.29, *p* > 0.05) at *T*_0_ between these two groups (see Table 1).

**Table 1:** Descriptive statistics and weight-status group differences for participants (*n* = 70)

	Baseline (*T*_0_)					5-year follow-up (*T*_1_)				
	NWG (*n* = 36) *M* (SD)/*N*(%)	OG (*n* = 34) *M* (SD)/*N*(%)	*t*/*χ*^2^	*p*	*d*	NWG (*n* = 36) *M* (SD)/*N* (%)	OG (*n* = 34) *M* (SD)/*N* (%)	*t*/*χ*^2^	*p*	*d*
*Z*-BMI	− 0.43 (0.70)	2.61 (0.83)	− 16.55	** < 0.001**	3.96	− 0.08 (1.00)	2.21 (1.39)	− 7.90	** < 0.001**	1.89
Age	10.65 (1.38)	10.58 (1.15)	0.24	0.811		15.80 (1.59)	16.52 (1.44)	− 1.98	0.052	
Gender			0.49	0.484				0.49	0.484	
Boys	15 (41.7)	17 (50)				15 (41.7)	17 (50)			
Girls	21 (58.3)	17 (50)				21 (58.3)	17 (50)			
SES	3.31 (0.71)	3.12 (1.07)	0.87	0.386		3.28 (0.91)	2.88 (1.23)	1.54	0.129	
Body esteem (BES/BESAA)*										
Total	20.56 (2.44)	10.53 (6.50)	8.64	** < 0.001**	2.05	2.62 (0.65)	1.83 (0.75)	4.64	** < 0.001**	1.11
Boys	20.60 (1.64)	9.12 (5.59)	7.66	** < 0.001**	2.77	2.60 (0.64)	1.99 (0.82)	2.30	**0.029**	0.82
Girls	20.52 (2.93)	11.94 (7.18)	5.00	** < 0.001**	1.56	2.63 (0.67)	1.69 (0.67)	4.29	** < 0.001**	1.40
Depressive symptoms (CDI)										
Total	5.89 (3.50)	9.65 (6.05)	− 3.20	**0.002**	0.72	8.28 (4.54)	12.94 (8.66)	− 2.84	**0.006**	0.67
Boys	7.47 (3.16)	9.53 (6.30)	− 1.15	0.261		8.27 (4.83)	11.53 (7.36)	− 1.46	0.155	
Girls	4.76 (3.35)	9.76 (6.00)	− 3.26	**0.002**	1.03	8.29 (4.44)	14.35 (9.82)	− 2.54	**0.016**	0.80
Depressive disorder			3.32	0.068				8.30	**0.004**	
Absence	36 (100)	31 (91.2)				34 (94.4)	23 (67.6)			
Risk/presence	0 (0)	3 (8.8)				2 (5.6)	11 (32.4)			

Significant *p* values are in bold. *NWG* normal-weight group, *OB* obesity group, *Z-BMI* body mass index *z*-scores, *SES* socioeconomic status, *BES* Body-Esteem Scale, *BESAA* Body-Esteem Scale for Adolescents and Adults, *M* mean, *SD* standard deviation. Cohen’s *d* was calculated for statistically significant associations: *d* < 0.4 = small effect, 0.4 ≤ *d* ≤ 0.75 = moderate effect, and *d* > 0.75 = large effect [54]

^*^ The BES (*T*_0_) and BESAA (*T*_1_) scales differ in the range of values they can take

### Measures

#### Body esteem

At *T*_0_, the Body-Esteem Scale (BES) [41, 42] was used. It is a 24-item self-reported scale (yes/no response) that collects information about feelings or perceptions about one’s appearance, weight, and the way participants believe they are valued by others. The total scores ranged from 0 to 24. In the current study, the BES scale demonstrated excellent reliability (*α* = 0.94).

At *T*_1_, the Body-Esteem Scale for Adolescents and Adults (BESAA) [14, 43] was used. The Spanish version consists of a self-administered questionnaire with 14 items on a 5-point Likert scale ranging from 0 (never) to 4 (always). It includes three subscales: BE-appearance, BE-weight, and BE-attribution. A total score is reported as the mean of the three subscale means (total score from 0 to 4), with high scores implying greater BE. Cronbach’s alpha for BESAA’s total score was 0.89.

#### Depressive symptoms and diagnosis

The Child Depression Inventory (CDI) [44, 45] was used at *T*_0_ and *T*_1_. This self-reported questionnaire consists of 27 items with three response options (0–2), aimed to measure cognitive, affective, and behavioral signs of depression. The total score of the scale ranged from 0 to 54. In the current study, Cronbach’s alpha for the CDI was 0.84 at *T*_0_ and 0.90 at *T*_1_.

The Schedule for Affective Disorders and Schizophrenia for School-Age Children-Present and Lifetime version (K-SADS-PL) [46, 47] was also used at *T*_0_ and *T*_1_. It is a semi-structured diagnostic interview to assess current and lifetime psychopathology. For the current study, we used a dichotomous variable to measure depressive disorder: (1) absence of a diagnosis and (2) presence of diagnosis (probable or confirmed clinical diagnosis).

#### Weight status

Trained interviewers measured height and weight using a Seca-digital weighing scale. Body Mass Index (BMI) was calculated (kg/m^2^), and BMI standard deviation scores (BMI *Z*-scores) were computed by comparing the child BMI with the BMI of the Spanish population of the same gender and age [48]. *Z*-BMI was categorized following the World Health Organization recommendations [49].

#### Age range

Age was calculated using the test date and the birth date reported by parents.

#### Socioeconomic-status (SES)

SES was determined by the Hollingshead Index [39], which considers education, occupation, gender, and marital status. It allows to obtain the place that a person occupies within the hierarchical structure of society ranging in level from 1 (lowest) to 5 (highest). To determine a child’s social status, scores for each primary caregiver are summed, and the total is divided by two.

### Strategy of analysis

#### Deviation from preregistration

The analysis reported here deviates on one issue from our preregistration plan. To reduce potential bias, multiple imputation (MI) of the missing values has been performed and MI complete case analyses have additionally been conducted. Strategies to handle missing data were not covered in the preregistration.

#### Preliminary analyses and data preparation

All analyses were performed in SPSS version 26.0. The Shapiro–Wilk test and plots showed some non-normal distributions for depressive symptoms and BE scores. Outliers were winsorized, after which plots showed no extreme outliers and values of skewness and kurtosis were within the normal range (CDI at *T*_0_: 1.19 and 1.11; CDI at *T*_1_: 1.06 and 0.53; BES at *T*_0_: − 0.14 and − 1.21; BESAA at *T*_1_: − 0.14 and − 1.21). Participants’ descriptive statistics and weight-status group differences for all variables were examined. Because very few children were assessed as being depressed, we focused on depressive symptoms measured by CDI instead of depression diagnosis in the regression model. Of note, CDI scores at *T*_1_ showed to be highly correlated with the presence of depression based on the diagnosis interview at *T*_1_ (*χ*^2^ = 0.81, *p* < 0.001). Pearson correlations between variables included in the tested model were analyzed.

The data were tested for missingness using mean comparisons, logistic regression, and Little’s MCAR test [50]. The results of the attrition analyses, reported in the Supplementary Tables S2 and S3, showed that there is no discernable pattern to the attrition in the data. We subsequently performed MI of the missing values on depressive symptoms at follow-up, using the FSC method in SPSS. Following the recommendation that the number of imputations should be at least equal to the percentage of cases with missing data [51], *m* = 30 imputed data sets were created, with *n* = 100 observations each.

#### Hypothesis testing

The first and second hypothesis were tested using Student’s *t* tests and multiple regression analysis, respectively. To test the hypotheses three and four on mediation and moderated mediation, models 4 and 58 of the Hayes PROCESS macro in SPSS were employed [52]. We performed the analyses with weight-status category at *T*_0_ as the primary independent variable (IV), and depressive symptoms at *T*_1_ as the dependent variable (DV). We entered BE at T_0_ as mediator in the model, and gender as a moderator of the effect of (*a* path) the IV on the mediator and (*b* path) the mediator on the DV (see Fig. 1). Mediation is determined by testing the significance of the indirect effect (*a* × *b*) of the IV on the DV through the mediator. Moderated mediation occurs when the indirect effect varies across values of the moderator (conditional effect). The *c′* path is the direct effect of the IV on the DV, controlled for the proposed moderated mediator effects. The 95% confidence intervals for the indirect effects were obtained using a 5000 bootstrap sample approach. Depressive symptoms at *T*_0_ were entered as a covariate to account for baseline symptoms. The effects were also controlled for age and SES.

The regression analyses and (moderated) mediation models were applied to both the subsample of participants with complete information and the imputed data sets with completed information. The MI estimates of the direct effects, their standard errors and associated *p* values, were obtained by pooling the parameters using Rubin’s rules [53]. To obtain bootstrap standard errors and 95% confidence intervals for the indirect effects, nonparametric bootstrapping was performed, with 5000 bootstrap samples drawn for each imputed data set, and the bootstrap inference results were thereupon pooled.

## Results

### Preliminary analyses

Descriptive statistics and weight groups’ differences for pre-imputed data are presented in Table 1. As can been seen, at both time points, the group with obesity showed higher scores on depressive symptoms (*T*_0_: *t* =  − 3.20, *p* = 0.002, *T*_1_: *t* =  − 2.84, *p* = 0*.*006) and lower scores on BE (*T*_0_: *t* = 8.64, *p* < 0.001, *T*_1_: *t* = 4.64, *p* < 0.001) compared to the normal-weight group. These results provide support for the *first hypothesis* of the current study. Note, however, that differences in depressive symptoms were statistically significant only for girls. Effect sizes (*d*) for all significant correlations ranged from 0.67 to 2.77, indicating moderate-to-large significant differences [54]. No significant differences were found for depression diagnosis at baseline, but the group with obesity had a significantly higher proportion of depression diagnoses at follow-up (*χ*^*2*^ = 8.30*, p* = 0.004). The correlations between all the variables included in the model are shown in Supplementary Table S4.

Subsequently, multiple regression analyses and analysis of (moderated) mediation were applied to both the complete and the completed (i.e., observed and imputed) data. As the results of the complete and the MI completed case analyses are rather similar, the latter are presented in the text (Tables 2, 3), and the former are displayed in the Supplementary material (Tables 5, 6).

**Table 2:** Pooled parameter estimates for the regression of depressive symptoms at *T*_1_ on weight status at *T*_0_ from completed (observed plus imputed) case analysis (*n* = 100, *m* = 30)

	B	SE	*p*
Constant	4.378	1.234	** < 0.001**
Weight status *T*_0_ (1 = obesity)	2.243	1.403	0.110
Depressive symptoms *T*_0_	0.695	0.140	** < 0.001**
*R*^*2*^			0.334

**Table 3:** Pooled parameter estimates of mediation and moderated mediation models from completed (observed plus imputed) case analysis (*n* = 100, *m* = 30)

	Mediation			Moderated mediation		
	*B*	SE	*p*	*B*	SE	*p*
Body esteem *T*_0_						
Constant	30.916	3.605	** < 0.001**	30.506	3.556	** < 0.001**
Gender (1 = boys)	− 0.798	0.835	0.342	0.866	1.172	0.462
Weight status *T*_0_ (1 = obesity)	− 8.061	0.851	** < 0.001**	− 6.721	1.074	** < 0.001**
Weight status *T*_0_ × gender				− 3.300	1.656	**0.049**
Depressive symptoms *T*_0_	− 0.504	0.081	** < 0.001**	− 0.522	0.080	** < 0.001**
Age *T*_0_	− 0.586	0.310	0.061	− 0.590	0.305	0.056
SES *T*_0_	− 0.116	0.484	0.812	0.133	0.477	0.780
* R*^*2*^	0.657			0.671		
Depressive symptoms *T*_1_						
Constant	13.439	7. 289	0.065	16.720	7.605	**0.028**
Gender	− 2.535	1.359	0.063	− 6.960	3.276	**0.034**
Weight status *T*_0_	− 0.359	1.943	0.853	− 0.358	1.928	0.853
Body esteem *T*_0_	− 0.337	0.170	**0.048**	− 0.465	0.198	**0.020**
Body esteem *T*_0_ × gender				0.281	0.192	0.144
Depressive symptoms *T*_0_	0.506	0.162	**0.002**	0.485	0.161	**0.003**
Age *T*_0_	0.237	0.532	0.656	0.167	0.526	0.751
SES *T*_0_	− 0.757	0.746	0.311	− 0.848	0.747	0.256
* R*^*2*^	0.415			0.433		

	Effect	SE	*p*	Effect	SE	*p*
Direct effects						
Weight status *T*_0_ on body esteem *T*_0_	− 8.061	0.851	** < 0.001**			
Girls				− 6.721	1.074	** < 0.001**
Boys				− 10.021	1.293	** < 0.001**
Body esteem *T*_0_ on depressive symptoms *T*_1_	− 0.337	0.170	**0.048**			
Girls				− 0.465	0.198	**0.020**
Boys				− 0.184	0.188	0.330
Weight status *T*_0_ on depressive symptoms *T*_1_	− 0.359	1.943	0.853	− 0.358	1.928	0.853

	Effect	Boot SE	Boot [LLCI, ULCI]*	Effect	Boot SE	Boot [LLCI, ULCI]*
Indirect effect						
Weight status *T*_0_ on depressive symptoms *T*_1_ via body esteem *T*_0_	2.717	1.434	[**0.016, 5.627**]			
Girls				3.124	1.390	[**0.490, 5.923**]
Boys				1.842	2.006	[− 1.923, 5.972]
Index of moderated mediation						
Gender				− 1.282	1.799	[− 4.654, 2.416]

Significant *p* values and bootstrap *95% CI* are in bold. Reference category for weight status *T*_0_ is normal weight, and for Gender girls

^*^ Lower and upper level of 95% bootstrap confidence interval

Table 2 shows the results of the regression of depressive symptoms at follow-up on weight-status at baseline, with depressive symptoms at *T*_0_ entered as a covariate to account for baseline symptoms.

The table provides evidence that the *second hypothesis* should be rejected. Controlled for baseline depressive symptoms, there appeared to be no significant difference in depressive symptoms at *T*_1_ between children with obesity at baseline and those with normal weight (*t* = 1.60, *p* = 0.110).

### Mediation and moderated mediation results

The mediation hypothesis 3 was tested using PROCESS model 4 by Hayes [52]. The results are displayed in Table 3.

**Fig. 2:**
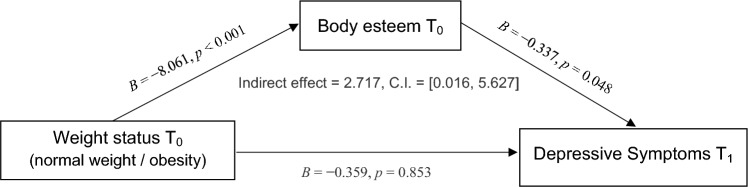
Diagram of mediation model results. Note that covariates (depressive symptoms, Age and SES at *T*_0_, and Gender) are included in the analyses but are not displayed in the figure

As can be seen, weight status had a significant negative effect on BE (*B* =  − 8.601, *p* < 0.001), and BE had a significant negative effect on depressive symptoms (*B* =  − 0.337, *p* = 0.048). The unconditional indirect effect of weight status on depressive symptoms through BE is positive (i.e., 2.717) and significantly different from zero (95% bootstrap *C.I.* [0.016, 5.627]). Hence, the *third hypothesis,* which argues that BE mediates the prospective association between childhood obesity and adolescent depressive symptoms, is supported. A graphical display of the mediation model results is provided in Fig. 2.

We subsequently tested the moderated mediation hypothesis 4 using PROCESS model 58 [52]. To facilitate interpretation, a bottom–up approach is used.

Analysis of *path a* showed no significant difference in BE between normal-weight boys and normal-weight girls (*B* = 0.866, *p* = 0.462). The interaction between weight status and gender, however, revealed a significant difference in BE between boys and girls with weight-status obesity, with boys with obesity having lower esteem than girls with obesity (*B* =  − 3.300, *p* < 0.049). Among girls, a significant negative association was found between weight status and BE (*B* =  − 6.721, *p* < 0.001). Among boys, the association between weight status and BE was *B* =  − 10.021 (*p* < 0.001). Hence, controlled for the other predictor variables, girls with obesity are predicted to score 6.721 lower on BE at baseline than normal-weight girls, whereas boys with obesity 10.021 lower than normal-weight boys. Examination of *path b* revealed a significant negative effect of BE on depressive symptoms at *T*_1_ among girls (*B* =  − 0.465, *p* < 0.020). This conditional effect for girls was not statistical different from the effect for boys (*B* = 0.281, *p* < 0.144). The direct effect of weight status on depressive symptoms at *T*_1_, corresponding to *path c′*, was also not significant.

With respect to mediation via BE and moderation by gender, the analysis revealed a significant positive indirect effect of weight status on depressive symptoms via BE of 3.124 for girls (95% bootstrap *C.I.* [0.490, 5.923]), and an insignificant effect of 1.842 for boys (95% bootstrap *C.I.* [− 1.923, 5.972]). The index of moderated mediation revealed that the difference between these conditional effects (i.e., − 1.282) was not significant (95% bootstrap *C.I.* [− 4.654, 2.416]). This implies that the mediation is not moderated by gender, so the *fourth hypothesis* should be rejected.

## Discussion

The current prospective study filled an eminent gap in the literature by investigating the mediating role of BE in explaining the childhood obesity–adolescent depressive symptoms link. In addition, potential gender moderation was examined. We found that the link between childhood obesity and follow-up depressive symptoms was mediated through BE. We did not find significant evidence of gender moderation for the full mediation model.

In line with prior evidence [6–8] and the concurrent part of our hypothesis, children with obesity reported higher rates of depressive symptoms at baseline and follow-up compared to children without obesity. However, in contrast to the hypothesis, no evidence of a direct prospective association between childhood obesity and later depressive symptoms was found. Previous studies have also reported inconsistent results [7, 12], and comparison between studies is hampered by differences in age of the target group and time of follow-up. Of note, the meta-analysis by Mannan et al. [5] noted a stronger prospective association between obesity and depressive symptoms for females in young adulthood than in adolescence, and among studies with longer follow-up periods (more than 10 years). A recent MR study found a causal relationship from childhood obesity to depression in adulthood [13]. Future research with longer term follow-up is needed to further increase insight into the link between childhood obesity and late adolescent depressive symptoms.

Whereas we did not find any evidence for the direct link between weight status and the development of depressive symptoms, we found that BE mediated this association, which is in line with our hypothesis. Thus, our results suggest that it is not obesity in childhood (i.e., weight status) per se, but the effect of weight status on BE, that plays a prominent role in whether children with obesity develop more depressive symptoms during adolescence. Although some previous cross-sectional studies have also found some support for the mediating role of BE [26–32], our study is unique by examining this link prospectively from childhood to adolescence. As such, our findings provide important implications for future preventive interventions in this stage. Specifically, targeting low BE among children with obesity may reduce later depressive symptoms during adolescence. In turn, derived behavioral changes (i.e., decreased sedentary lifestyle, less emotional eating) may reduce obesity [55], helping to prevent the vicious cycle of obesity and depressive symptomatology. Our results revealed a significant indirect effect of weight status on depressive symptoms via BE for girls but not for boys. However, the index of moderated mediation was not significant, which means that the difference in indirect effects between boys and girls is not significantly different [52]. And this implies that BE-mediating effects do not differ by gender. Of note, one previous cross-sectional study found a mediation effect of BE among adolescent girls, but not boys [33]. It is possible that gender differences regarding the mediating effect of BE become more important when children become older. Further research is needed to examine this potential relationship.

Although BE mediation did not differ between boys and girls, the specific link between obesity and BE was stronger in boys compared to girls. Boys with obesity had lower BE compared to girls. This is a remarkable finding, as previous studies mostly reported similar associations between obesity and BE for boys and girls [18, 34]. An explanation for this finding might be that children with obesity are generally limited in doing sports, while these sport activities prove to be particularly important for body image among boys [56]. Moreover, McCabe et al. [57] pointed out that boys received more messages aimed at muscle gain. In this sense, BE refers to a nuanced concept that goes beyond weight status [14] and might also reflect the muscular ideal, which is particularly internalized by boys [58]. Future studies should further explore whether there are specific components of BE (i.e., weight, appearance) that are influenced by weight status. Finally, in contrast to our hypothesis, we found no evidence that gender moderated the association between BE and the development of depressive symptoms at follow-up. This finding is in line with some other studies [22, 37, 38], but there are also studies suggesting stronger links among females compared to males. One possible explanation relates to the increasing pressures regarding body ideals at younger ages also among boys [59].

### Strengths, limitations, and future directions

Our study is based on a case–control design of children with and without obesity who were matched on important sociodemographic characteristics (i.e., SES, age) and who were followed over time. Our prospective design and the matching are notable strengths. However, there are also some limitations that should be acknowledged. First, although we prospectively examined the development of depressive symptoms over time, the IV and the mediator were assessed at the same time point. For further studies, it would be relevant to test for competing explanations, such as the role of weight bias and weight teasing on BE and depressive symptoms [60]. Moreover, our sample comes from a specific region of Spain. This may limit the generalizability of the results. Finally, while our sample size is relatively small, the case–control design lowered the need to control for various covariates. Moreover, we have imputed missing data and findings were rather similar for complete and completed (i.e., observed and imputed) case analyses. Nevertheless, future studies are encouraged to recruit larger, representative samples. To help guide sample size decisions for such a study, post hoc Monte Carlo power analysis for the unconditional indirect effect in this study, with sample size set to *n* = 100 and *α* set to 0.05, revealed an acceptable power of 0.76 [61]. Following Aberson et al. [62], to achieve similar statistical power for the conditional indirect effects in the moderated mediation model, a sample size of 2*n* is recommended.

## Conclusions

The current study provides evidence that BE mediates the prospective association between childhood obesity and adolescent depressive symptoms. We did not find significant evidence that gender moderates this mediation. Future studies with larger sample sizes and longer term follow-ups are urged to re-examine this moderated mediation. The findings may have important implications for clinical practice. Future clinical preventive intervention trials should examine whether improving BE in high-risk children (i.e., children with obesity) reduces the development of adolescent depressive symptoms.

### Supplementary Information

Below is the link to the electronic supplementary material.

Supplementary file1 (DOCX 52 KB)

## Data Availability

The datasets generated during and/or analyzed during the current study are available from the corresponding author on reasonable request.
