# Supplementary material for: Childhood obesity and adolescent follow-up depressive symptoms: exploring a moderated mediation model of body esteem and gender
Source: Eur Child Adolesc Psychiatry. 2024 Feb 7;33(8):2859–69. doi: 10.1007/s00787-023-02348-9 (PMC11272700; doi:10.1007/s00787-023-02348-9)
Supplement: Supplementary file 1 — Supplementary file1 (DOCX 52 KB) [file 787_2023_2348_MOESM1_ESM.docx]

**Supplement**

**Table S1** Descriptive statistics and weight group differences for the total sample (*n* = 100) at T_0_

|  | **Baseline (T_0_)** | | | | |
| --- | --- | --- | --- | --- | --- |
|  | **NWG**  **(n = 50)** | **OG**  **(*n* = 50)** | **Group Difference** | | |
|  | *M (SD)* /N(%) | *M (SD)/* N(%) | *t / χ²²* | *p* | *d* |
| Z-BMI | −0.39 (0.68) | 2.83 (0.95) | −19.44 | **< 0.001** | 3.90 |
| Age | 10.70 (1.46) | 10.53 (1.21) | 0.62 | 0.536 |  |
| Gender |  |  | 0.04 | 0.838 |  |
| Boys | 19 (40) | 20 (40) |  |  |  |
| Girls | 31 (60) | 30 (60) |  |  |  |
| SES | 3.20 (0.76) | 3.12 (0.94) | 0.47 | 0.640 |  |
| Body esteem (BES) |  |  |  |  |  |
| Total | 20.58 (2.52) | 11.16 (6.42) | 9.66 | **< 0.001** | 1.93 |
| Boys | 20.74 (2.28) | 10.15 (6.06) | 7.14 | **< 0.001** | 2.31 |
| Girls | 20.48 (2.68) | 11.83 (6.66) | 6.70 | **< 0.001** | 1.70 |
| Depressive symptoms (CDI) |  |  |  |  |  |
| Total | 6.74 (3.82) | 9.62 (6.14) | −2.82 | **0.006** | 0.56 |
| Boys | 7.47 (3.03) | 8.85 (6.28) | −0.87 | 0.393 |  |
| Girls | 6.29 (4.21) | 10.13 (6.11) | −2.87 | **0.006** | 0.73 |
| Depressive disorder |  |  | 3.84 | 0.050 |  |
| Absence | 49 (98) | 44 (88) |  |  |  |
| Risk/presence | 1 (2) | 6 (12) |  |  |  |

Significant *p*-values are in bold. To obtain *p*-values, tail probabilities of Chi-square distribution were used for categorical variables and Student’s *t*-distribution for continuous variables. Abbreviations: NWG = Normal-Weight group, OB = Obesity group, Z-BMI = Body Mass Index z-scores, SES = Socioeconomic Status, BES = Body Esteem Scale, BESAA = Body Esteem Scale for Adolescents and Adults, M = Mean, SD = Standard Deviation.

**Table S2** Frequencies and mean comparisons of participants and non-participants at T_0_ (*n* = 100)

|  | **Participants**  **(*n* = 70)** | **Non-participants (*n* = 30)** |  | | |
| --- | --- | --- | --- | --- | --- |
|  | N (%) / M (SD) | N (%) / M (SD) | *t / χ²* | | *p* |
| Weight-status T_0_ |  |  | 0.19 | 0.663 | |
| Normal Weight | 36 (72.0) | 14 (28.0) |  |  | |
| Obesity | 34 (68.0) | 16 (32.0) |  |  | |
| Gender |  |  | 4.42 | **0.035** | |
| Boys | 32 (82.1) | 7 (17.9) |  |  | |
| Girls | 38 (62.3) | 23 (37.7) |  |  | |
| SES T_0_ | 3.21 (0.90) | 3.03 (0.72) | −0.98 | 0.332 | |
| Age T_0_ | 10.61 (1.26) | 10.60 (1.52) | −0.05 | 0.960 | |
| Body esteem T_0_ | 15.69 (6.98) | 16.30 (6.38) | 0.41 | 0.680 | |
| Depressive symptoms T_0_ | 7.71 (5.23) | 9.27 (5.37) | 1.35 | 0.180 | |

Significant *p*-values are in bold. To obtain *p*-values, tail probabilities of Chi-square distribution were used for categorical variables and Student’s *t*-distribution for continuous variables.

**Table S3** Logistic regression of participation vs. non-participation at follow-up (T_1_) on participant characteristics (*n* = 100)*

|  |  | ***B*** | ***SE*** | ***p*** | ***Exp(B)*** |  |
| --- | --- | --- | --- | --- | --- | --- |
| Constant |  | 3.082 | 2.693 | 0.252 | 21.796 |  |
| Gender (1 = Boys) |  | 0.956 | 0.507 | 0.059 | 2.602 |  |
| Weight status T_0_ (1 = Obesity) |  | −0.830 | 0.655 | 0.205 | 0.436 |  |
| Body esteem T_0_ |  | −0.106 | 0.059 | 0.073 | 0.899 |  |
| Depressive symptoms T_0_ |  | −0.110 | 0.055 | **0.046** | 0.896 |  |
| Age T_0_ |  | −0.027 | 0.175 | 0.878 | 0.974 |  |
| SES T_0_ |  | 0.247 | 0.281 | 0.379 | 1.280 |  |
|  |  |  |  |  |  |  |
| -2*LL* model |  |  |  |  | 111.411 |  |
| -2*LL* intercept-only model |  |  |  |  | 122.173 |  |
|  |  |  |  |  |  |  |

Significant *p*-values are in bold.

* According to the likelihood ratio test, the null hypothesis that the *B* coefficients of the predictors are all equal to zero cannot be rejected at the 0.05 level (*χ*² = 10.961, *df* = 6, *p* = 0.096). Further, Little’s MCAR test [51] leads to the conclusion that we cannot reject the null that the unobserved data are missing completely at random (MCAR) (*χ*² = 10.363, *df* = 6, *p* = 0.110).

**Table S4** Correlations between study variables and their significance levels (*n* = 100)*

|  | 1 | 2 | 3 | 4 | 5 | 6 | 7 |
| --- | --- | --- | --- | --- | --- | --- | --- |
| 1. Depressive symptoms T_1_ ^a^ |  | -0.15 | **0.30** | **-0.51** | **0.56** | 0.12 | −0.11 |
| 2. Gender (1 = Boys) ^b^ | 0.166 |  | 0.02 | −0.07 | 0.00 | −0.03 | −0.01 |
| 3. Weight status T_0_ (1 = Obesity) ^c^ | **0.005** | 0.840 |  | **−0.70** | **0.27** | −0.06 | −0.05 |
| 4. Body esteem T_0_ ^d^ | **<0.001** | 0.510 | **<0.001** |  | **−0.57** | −0.12 | 0.03 |
| 5. Depressive symptoms T_0_ ^e^ | **<0.001** | 0.999 | **0.006** | **<0.001** |  | 0.10 | −0.05 |
| 6. Age T_0_ ^e^ | 0.314 | 0.786 | 0.536 | 0.252 | 0.341 |  | 0.08 |
| 7. SES T_0_ ^e^ | 0.302 | 0.954 | 0.640 | 0.807 | 0.627 | 0.455 |  |

Correlations are above and their *p*-values are below the main diagonal. Significant *p*-values are in bold.

* The figures for Depressive symptoms T_1_ are multiple imputation (*m* = 30) pooled correlations (top row) and pooled *p*-values (left-most column). The other correlations and *p*-values are not pooled.

^a^ Outcome, ^b^ Moderator, ^c^ Primary predictor, ^d^ Mediator, ^e^ Covariate.

**Table S5** Parameter estimates for the regression of depressive symptoms at T_1_ on weight status at T_0_ from complete case analysis (*n* = 70)

|  |  |  | ***B*** | ***SE*** | ***p*** |
| --- | --- | --- | --- | --- | --- |
| Constant |  |  | 4.277 | 1.336 | **< 0.001** |
| Weight status T_0_ (1 = Obesity) |  |  | 2.110 | 1.549 | 0.178 |
| Depressive symptoms T_0_ |  |  | 0.679 | 0.149 | **< 0.001** |
| *R^2^* |  |  |  |  | 0.318 |

**Table S6** Parameter estimates of moderated mediation and mediation model from complete case analysis (*n* = 70)

|  |  | **Mediation** | | |  | | **Moderated Mediation** | | | |
| --- | --- | --- | --- | --- | --- | --- | --- | --- | --- | --- |
|  |  | ***B*** | ***SE*** | ***p*** | |  | | ***B*** | ***SE*** | ***p*** |
| **Body esteem T_0_** |  |  |  |  | |  | |  |  |  |
| Constant |  | 29.869 | 4.533 | **< 0.001** | |  | | 29.190 | 4.394 | **<0.001** |
| Gender (1 = Boys) |  | −0.876 | 1.014 | 0.391 | |  | | 1.405 | 1.387 | 0.315 |
| Weight status T_0_ (1 = Obesity) |  | −8.049 | 1.070 | **< 0.001** | |  | | −5.836 | 1.406 | **<0.001** |
| Weight status T_0_ × Gender |  |  |  |  | |  | | −4.542 | 1.952 | **0.023** |
| Depressive symptoms T_0_ |  | −0.511 | 0.104 | **< 0.001** | |  | | −0.546 | 0.101 | **<0.001** |
| Age T_0_ |  | −0.604 | 0.403 | 0.138 | |  | | −0.608 | 0.389 | 0.123 |
| SES T_0_ |  | 0.151 | 0.566 | 0.791 | |  | | 0.144 | 0.547 | 0.793 |
| *R^2^* |  | 0.673 | | |  | | 0.600 | | | |
| **Depressive symptoms T_1_** |  |  |  |  | |  | |  |  |  |
| Constant |  | 14.400 | 8.322 | 0.088 | |  | | 18.002 | 8.591 | **0.040** |
| Gender |  | −2.573 | 1.446 | 0.080 | |  | | −7.365 | 3.525 | **0.041** |
| Weight status T_0_ |  | −0.821 | 2.082 | 0.695 | |  | | −0.624 | 2.066 | 0.764 |
| Body esteem T_0_ |  | −0.357 | 0.177 | **0.048** | |  | | −0.512 | 0.204 | **0.015** |
| Body esteem T_0_ × Gender |  |  |  |  | |  | | 0.308 | 0.207 | 0.142 |
| Depressive symptoms T_0_ |  | 0.530 | 0.172 | **0.003** | |  | | 0.496 | 0.172 | **0.005** |
| Age T_0_ |  | 0.149 | 0.581 | 0.798 | |  | | 0.088 | 0.577 | 0.880 |
| SES T_0_ |  | −0.732 | 0.802 | 0.365 | |  | | −0.796 | 0.796 | 0.321 |
| *R^2^* |  | 0.393 | | |  | | 0.414 | | | |
|  |  | ***Effect*** | ***SE*** | ***p*** | |  | | ***Effect*** | ***SE*** | ***p*** |
| **Direct effect** |  |  |  |  | |  | |  |  |  |
| Weight status T_0_ on Body esteem T_0_ |  | −8.049 | 1.070 | **< 0.001** | |  | |  |  |  |
| Girls |  |  |  |  | |  | | −5.836 | 1.406 | **<0.001** |
| Boys |  |  |  |  | |  | | −10.378 | 1.440 | **<0.001** |
| Body esteem T_0_ on Depressive symptoms T_1_ |  | −0.357 | 0.177 | **0.048** | |  | |  |  |  |
| Girls |  |  |  |  | |  | | −0.512 | 0.204 | **0.015** |
| Boys |  |  |  |  | |  | | −0.204 | 0.203 | 0.320 |
| Weight status T_0_ on Depressive symptoms T_1_ |  | −0.821 | 2.082 | 0.695 | |  | | −0.624 | 2.066 | 0.764 |
|  |  | ***Effect*** | ***Boot SE*** | ***Boot [LLCI, ULCI]**** | |  | | ***Effect*** | ***Boot SE*** | ***Boot [LLCI, ULCI]**** |
| **Indirect effect** |  |  |  |  | |  | |  |  |  |
| Weight status T_0_ on Depressive symptoms T_1_ via Body esteem T_0_ | | 2.875 | 1.521 | **[0.039, 5.962**] | |  | |  |  |  |
| Girls |  |  |  |  | |  | | 2.988 | 1.322 | [**0.570, 5.803**] |
| Boys |  |  |  |  | |  | | 2.118 | 2.289 | [−2.124, 6.883] |
|  |  |  |  |  | |  | |  |  |  |
| **Index of moderated mediation** |  |  |  |  | |  | |  |  |  |
| Gender |  |  |  |  | |  | | −0.870 | 2.151 | [−4.893, 3.693] |
|  |  |  |  |  | |  | |  |  |  |

Significant *p*-values are in bold. Reference category for Weight status T_0_ is normal weight, and for Gender girls.

^*^ Lower and upper level of 95% bootstrap confidence interval.
